# Supplementary figures and images for: SARS-CoV-2-specific T cells associate with inflammation and reduced lung function in pulmonary post-acute sequalae of SARS-CoV-2
Source: PLoS Pathog. 2022 May 26;18(5):e1010359. doi: 10.1371/journal.ppat.1010359 (PMC9176759; doi:10.1371/journal.ppat.1010359)

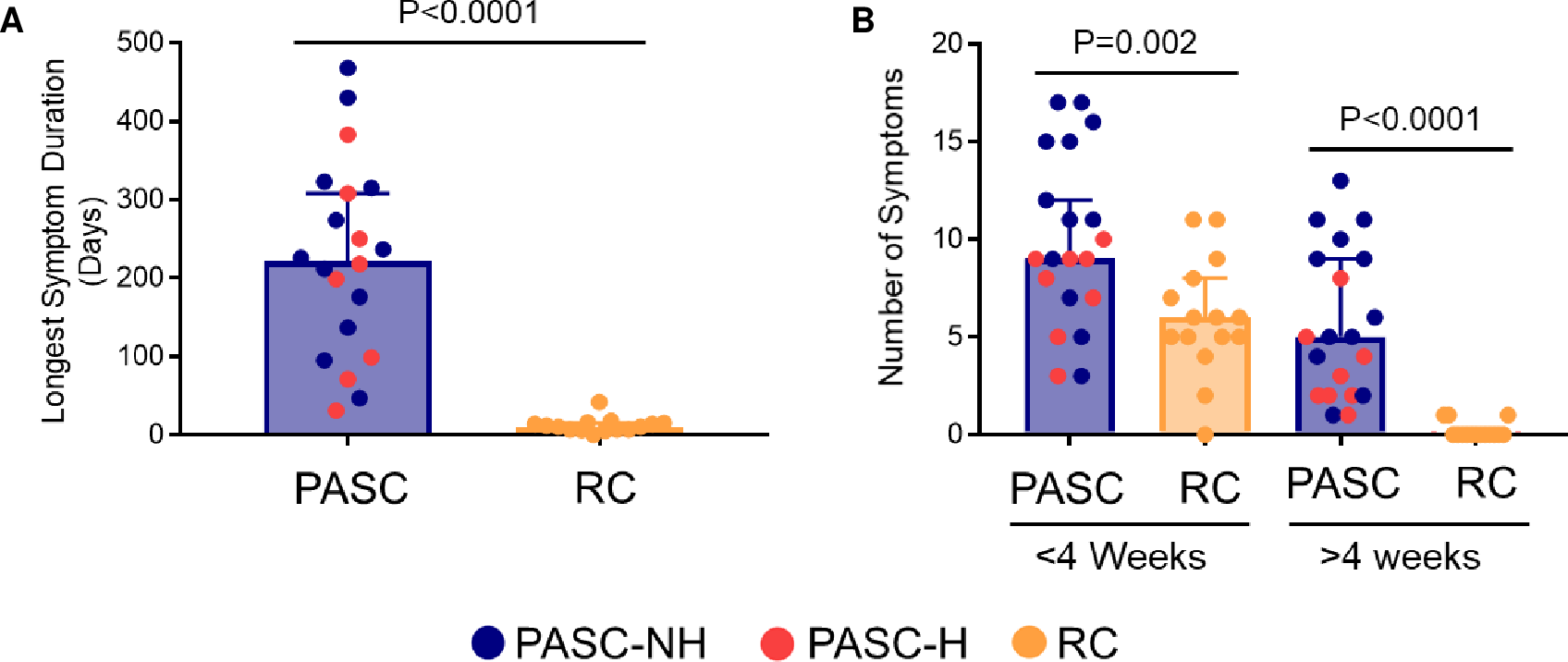

Supplement: S1 Fig — (A) Symptom duration (days) reported in symptom questionnaires for PASC and RC participants. (B) Number of symptoms reported <4 weeks or >4 weeks from symptom onset for PASC and RC participants. For each graph, bars represent the median of each cohort and the error bar represents the upper 95% confidence interval. Blue represents PASC participants not hospitalized (PASC-NH, n = 12), red represents PASC-hospitalized (PASC-H, n = 8) and orange represents RC participants (n = 20). Mann-Whitney tests were used to determine statistical significance. (TIF) [file ppat.1010359.s003.tif]

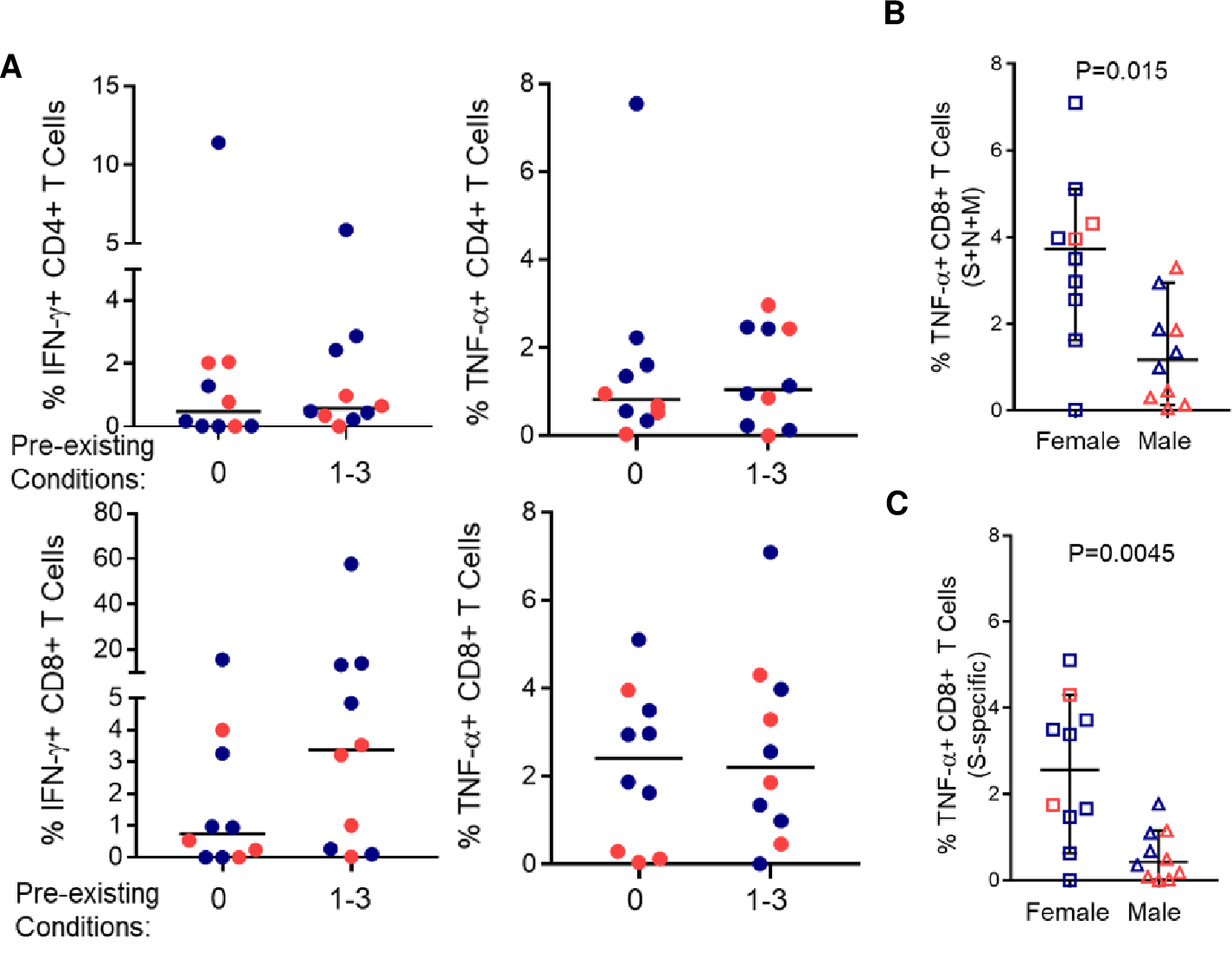

Supplement: S2 Fig — (A) Percent of combined S-, N- and M-specific frequencies of IFN-γ- or TNF-α-producing CD4+ or CD8+ T cells separated by number of pre-existing conditions. (B) Frequency of combined S-, N- and M-specific TNF-α-producing CD8+ T cells or (C) S-specific TNF-α-producing CD8+ T cells for PASC participants separated by sex. Each point represents data from one participant where blue represents PASC-NH (not hospitalized) and red represents PASC-hospitalized. Mann-Whitney tests were used to determine statistical significance. (TIF) [file ppat.1010359.s004.tif]

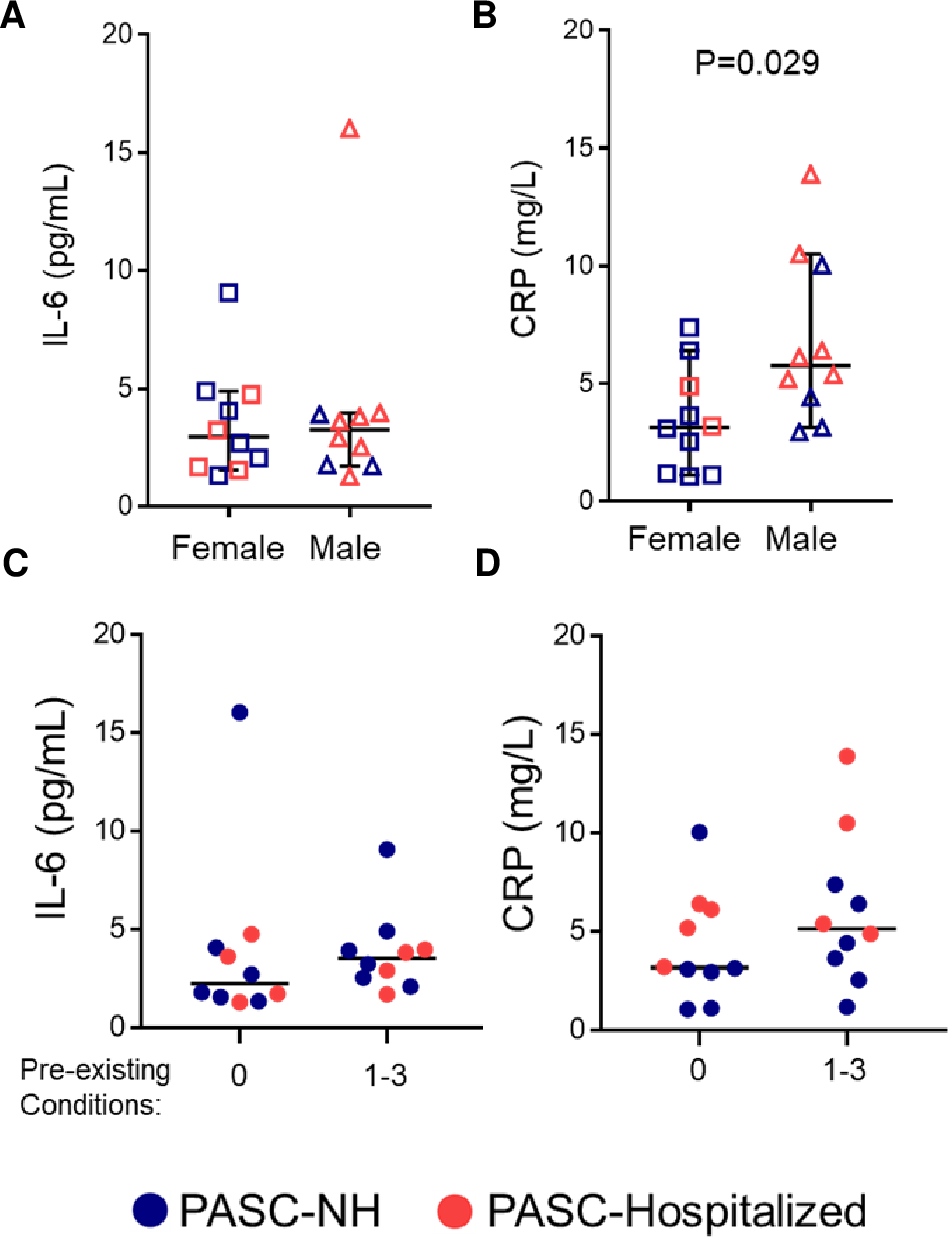

Supplement: S3 Fig — (A) PASC plasma IL-6 (pg/mL) levels and (B) PASC plasma CRP levels (mg/L) in PASC separated by sex. (C) Serum IL-6 (pg/mL) and (D) CRP (mg/L) in PASC separated by pre-existing conditions. Each point represents data from one participant. Where applicable, bar represents median of cohort and error bars are 95% confidence index. Blue and red symbols represent PASC-NH (not hospitalized) and PASC-Hospitalized, respectively. (TIF) [file ppat.1010359.s005.tif]

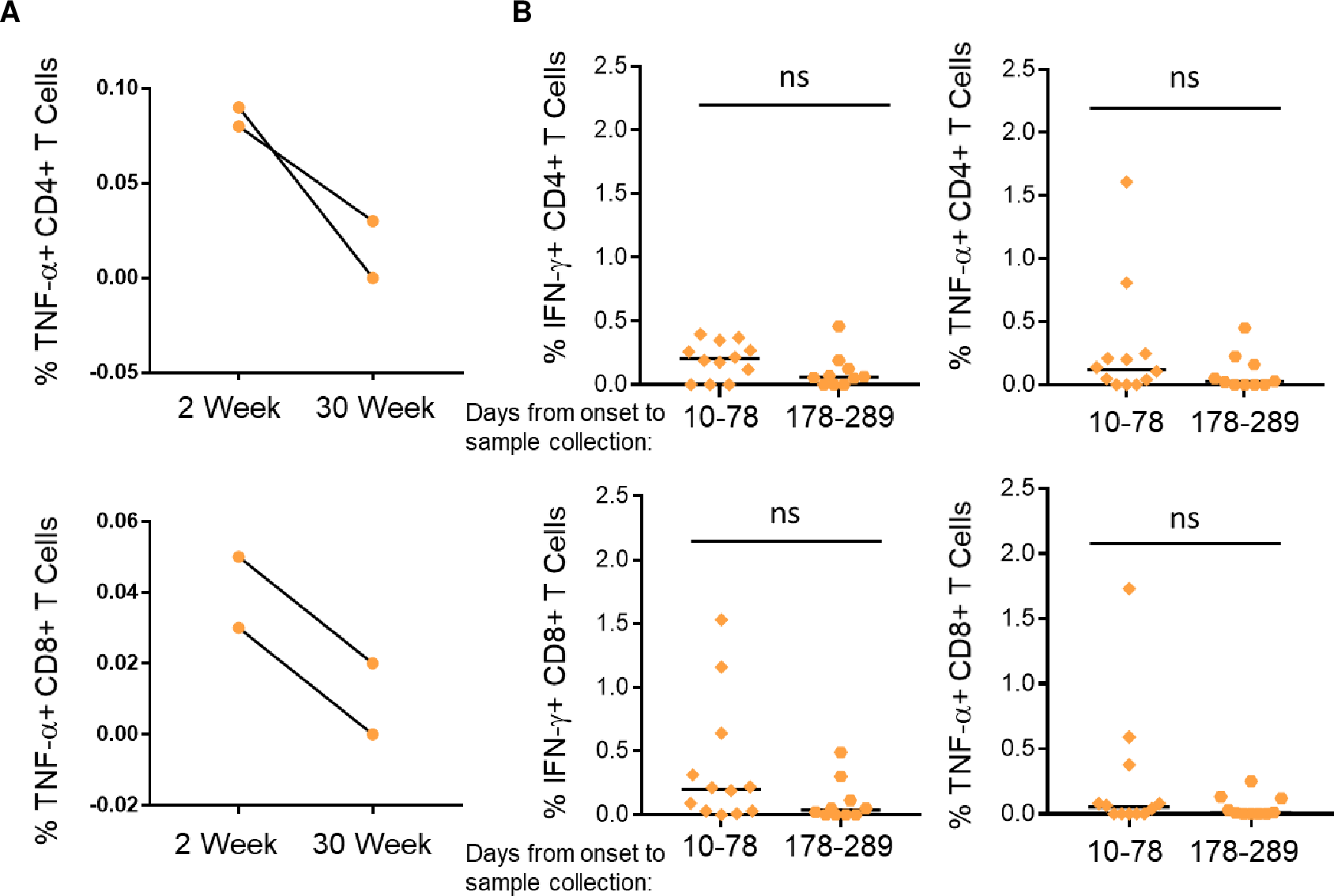

Supplement: S4 Fig — (A) Frequency of SARS-CoV-2 N- and M-specific TNF-α-producing T cells from samples taken 2 and 30 weeks from symptom onset. Each set of points connected by a line represents data from one participant. (B) Percent of cummulative S, N and M SARS-CoV-2-specific CD4+ or CD8+ T cells producing IFN-γ or TNF-α for the RC cohort split by time from symtom onset to sample collection. Orange symbols represent RC participants. (TIF) [file ppat.1010359.s006.tif]

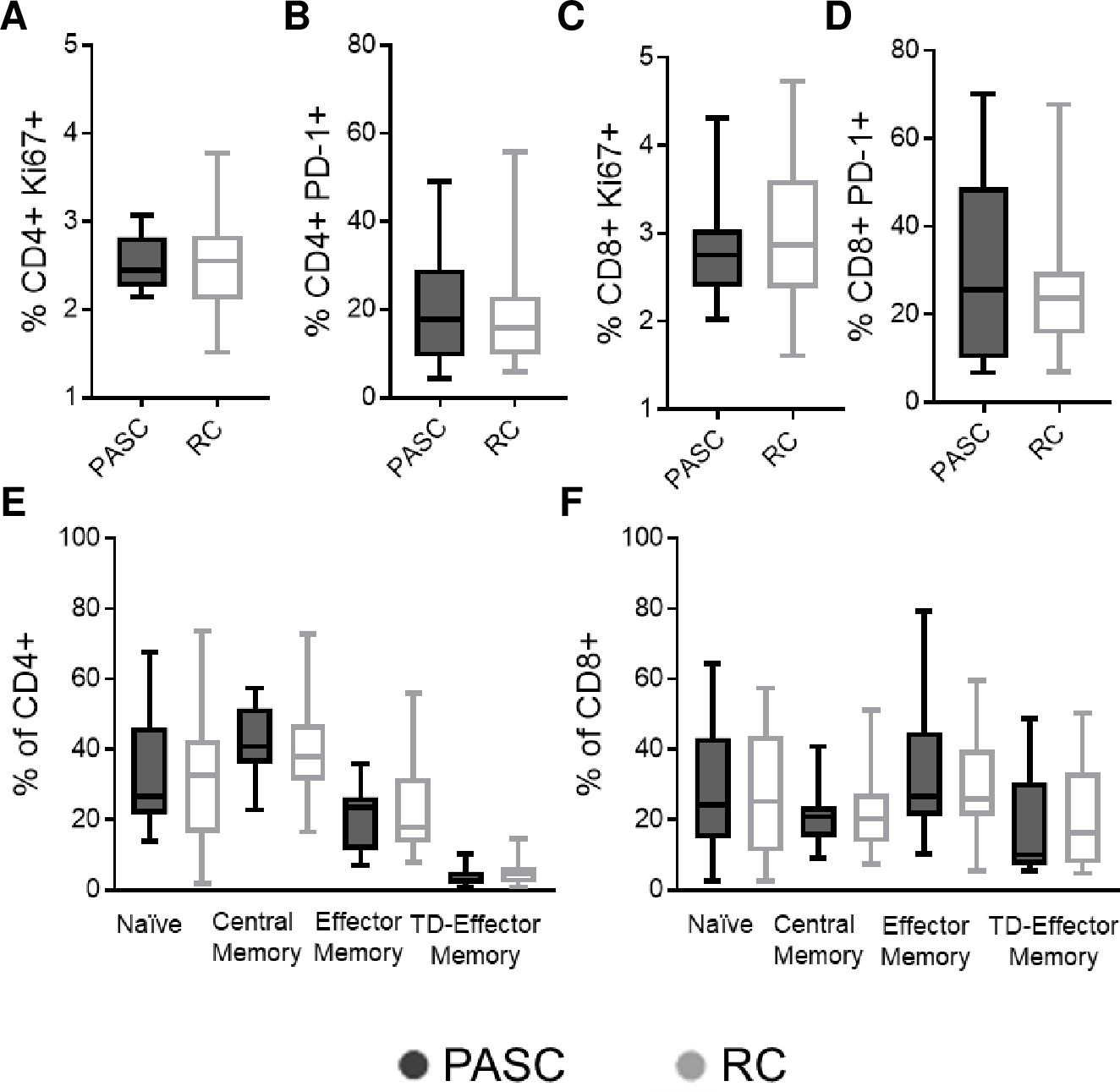

Supplement: S5 Fig — PBMCs were isolated from PASC and RC participants and stained with mAb to assess their ex vivo phenotype by flow cytometric analysis. Percentage of total CD4+ T cells expressing (A) Ki-67 and (B) PD-1 for PASC and RC participants. Percentage of total CD8+ T cells expressing (C) Ki-67 and (D) PD-1 for both cohorts. Shown are the frequencies of naïve (CD27+CD45RA+), central memory (CD27+CD45RA-), effector memory (CD27-CD45RA-) and terminally differentiated (TD)-effector memory (CD27-CD45RA+) on total (E) CD4+ and (F) CD8+ T cells. All data collected from flow cytometry in unstimulated conditions. For each box and whisker plot, the center line denotes the median value (50th percentile), and the box contains the 25th to 75th percentile values for each dataset. Whiskers mark the 5th and 95th percentiles, and values beyond these upper and lower bounds are not visualized. Dark grey plots represent the PASC cohort and light gray plots represent the RC cohort. Mann-Whitney tests were used to determine statistical significance. (TIF) [file ppat.1010359.s007.tif]

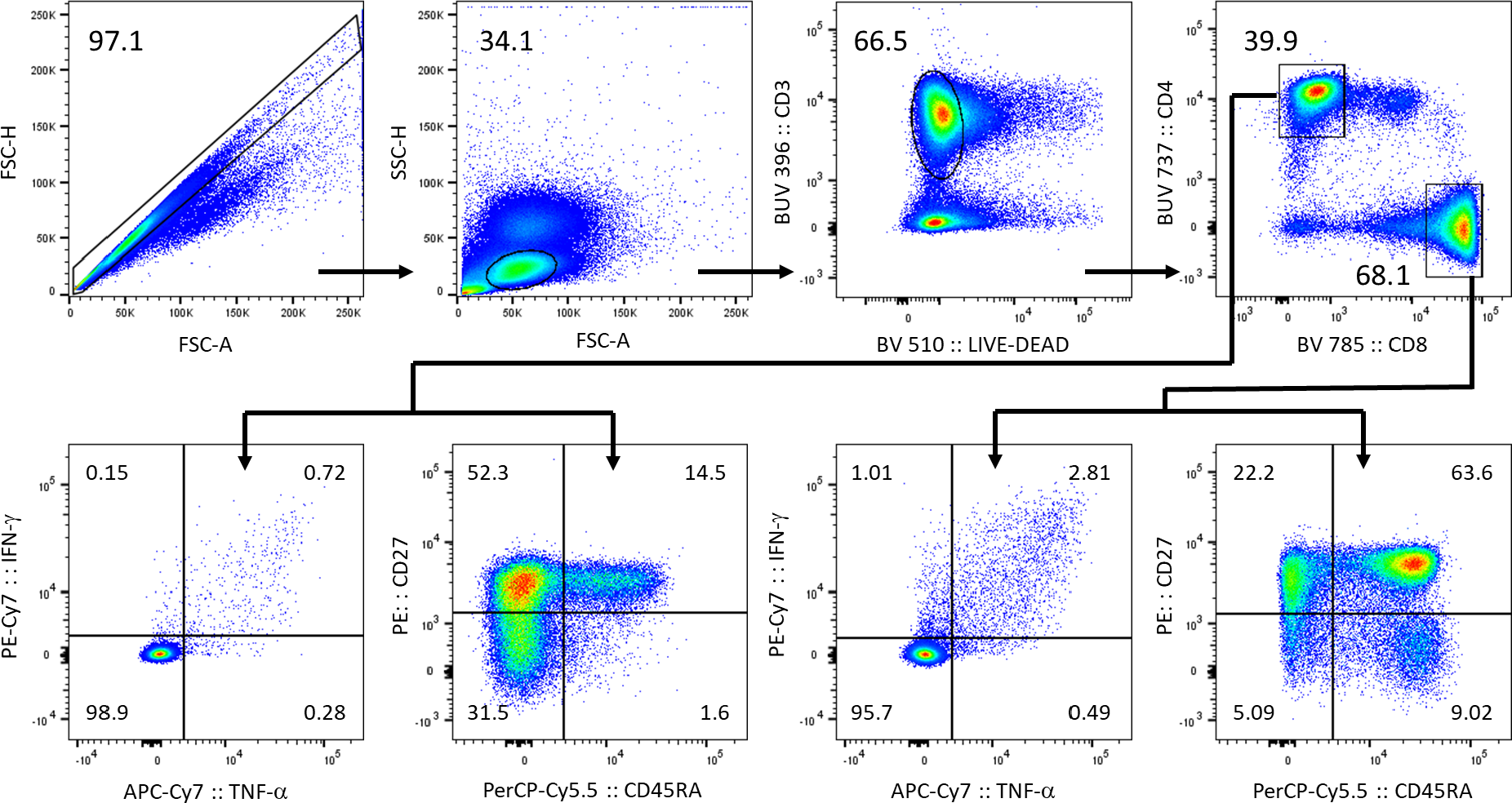

Supplement: S6 Fig — An example of the gating strategy used to determine frequency of SARS-CoV-2-specific T cells. Each axis is labeled with the fluorochrome measured and corresponding marker if applicable and data is presented as pseudocolor dot plots (FlowJo). Arrows indicate the sequence of analysis and the number on each plot is the frequency of the gated population. (TIF) [file ppat.1010359.s008.tif]
